# Associations of serum lipid traits with DLBCL: a prospective cohort study from the UK Biobank

**DOI:** 10.3389/fnut.2026.1707450

**Published:** 2026-02-10

**Authors:** QingQing Luo, ShanShan Cai, LinHui Hu, Ya Wang, Li Yu

**Affiliations:** Department of Hematology, The Second Affiliated Hospital, Jiangxi Medical College, Nanchang University, Jiangxi Provincial Key Laboratory of Hematological Diseases, Nanchang, Jiangxi, China

**Keywords:** apolipoproteins A, diffuse large B-cell lymphoma, high-density lipoprotein, serum lipids, UK Biobank

## Abstract

**Background:**

Diffuse large B-cell lymphoma (DLBCL) is the most common subtype of non-Hodgkin lymphoma (NHL), accounting for approximately 30% of all NHL cases. While serum lipids have been associated with various cancers, their relationship with the risk of DLBCL remains largely unexplored.

**Methods:**

This prospective cohort study included 339,172 participants from the UK Biobank. Baseline serum levels of apolipoproteins A and B (ApoA/B), high-and low-density lipoprotein cholesterol (HDL/LDL), total cholesterol (TC), and triglycerides (TG) were measured. The associations between lipid profiles and DLBCL risk were assessed using Cox proportional hazards models, and restricted cubic spline (RCS) analysis. Subgroup analyses and temporal lipid trajectories were also performed.

**Results:**

Over a median follow-up of 13.8 years, 1,207 participants developed DLBCL. Lower levels of ApoA, HDL, and TC were significantly associated with increased DLBCL risk. RCS analysis revealed non-linear associations for ApoA and HDL, and a linear association for TC (P for non-linearity: 0.048, 0.017, and 0.139, respectively). Subgroup analysis indicated a significant interaction with age. Temporal trajectory analysis showed a gradual decline in ApoA and HDL levels during the 10 years prior to diagnosis, with a steeper drop in the last 5 years.

**Conclusion:**

Reduced levels of ApoA, HDL, and TC are linked to a higher risk of DLBCL. Notably, lipid changes precede clinical diagnosis by several years, suggesting their potential as early indicators for DLBCL risk stratification and preventive strategies.

## Introduction

Diffuse large B-cell lymphoma (DLBCL) is the most common subtype of non-Hodgkin lymphoma (NHL), accounting for approximately 30% of NHL cases. Despite significant advances in understanding the pathophysiological mechanisms of DLBCL in recent years, its exact etiology remains unclear (1). Serum lipids are not only key components of cell membranes but also play essential roles in various biological processes, including inflammation, cell signal transduction, and immune regulation (2). Recent studies have shown that lipid metabolism directly contributes to tumorigenesis by regulating cell proliferation, apoptosis, and immune evasion, as well as by remodeling the tumor microenvironment (3, 4). Therefore, a systematic investigation into the relationship between serum lipid profiles and DLBCL risk is crucial not only for elucidating the potential mechanisms of the disease but also for providing new insights into the development of lipid metabolism-targeted intervention strategies.

However, clinical studies on the relationship between serum lipids and DLBCL have shown significant controversy. One study found that reduced levels of total cholesterol (TC), high-density lipoprotein cholesterol (HDL-C), and low-density lipoprotein cholesterol (LDL-C) were significantly associated with poorer progression-free survival (PFS) and overall survival (OS) in DLBCL (5). Another study indicated that triglyceride (TG) levels had a negative impact on PFS (*P* = 0.013), while HDL-C levels had a protective effect on OS (*P* = 0.003) (6). Furthermore, a study reported that low levels of serum apolipoprotein A1 (ApoA1) were an independent adverse prognostic factor for OS, although no significant correlation was observed with PFS (7). Our previous research also revealed that DLBCL patients had significantly lower serum levels of TG, LDL-C, HDL-C, ApoA-I, and ApoB compared to healthy controls, with these levels significantly increasing after chemotherapy (8). However, these studies are often limited by small sample sizes, cross-sectional designs, and a focus on single lipid markers. Moreover, while most current research has concentrated on the impact of serum lipids on the prognosis of DLBCL, studies exploring the relationship between lipids and the risk of DLBCL remain relatively scarce.

The UK Biobank, a large prospective cohort integrating multidimensional clinical data, genetic information, and biochemical markers, provides an ideal platform for investigating the relationship between serum lipid profiles and the risk of DLBCL. This study is the first large-scale, prospective, and systematic research on the association between lipid traits and DLBCL risk. Its goal is to examine the changes in lipid profiles associated with the onset of DLBCL, providing new theoretical insights for the development of targeted prevention strategies.

## Methods

### Study population

The UK Biobank is a community-based prospective cohort study that includes approximately 500,000 individuals aged between 40 and 69 years, who were recruited between 2006 and 2010. Potential participants were required to visit one of 22 assessment centers across England, Wales, and Scotland, where they underwent physical examinations, provided biological samples, and completed baseline questionnaires. Detailed information regarding the study procedures is available in other publications (9). After excluding individuals with missing lipid data (*n* = 162,767) and those diagnosed with DLBCL at baseline (*n* = 192), a total of 339,172 individuals were included in the final analysis (Supplementary Figure 1).

UK Biobank was constructed under ethical approval obtained by the North West Multi-Centre Research Ethics Committee (REC reference: 11/NW/03820) and all participants provided written informed consent prior to participation. The current analyses were carried out under Application Number 290648.

### Data collection

In the UK Biobank study, researchers conducted a comprehensive assessment of serum lipid profiles using blood samples collected during participants' initial registration, including apolipoprotein A (ApoA), apolipoprotein B (ApoB), total cholesterol (TC), high-density lipoprotein cholesterol (HDL), low-density lipoprotein cholesterol (LDL), and triglycerides (TG). These lipid traits were quantitatively measured using immunoturbidimetry on a Beckman Coulter AU5800, an automated blood analyzer. Apolipoproteins were measured in grams per liter (g/L), while cholesterol and triglycerides were measured in millimoles per liter (mmol/L). The UK Biobank provides detailed descriptions of the methods for processing these serum samples and conducting the necessary assays (10).

We used the touchscreen questionnaire to derive information on several potential confounders: age, sex, ethnicity, Body Mass Index (BMI), Townsend Deprivation Index (TDI), smoking and alcohol consumption status, education level, physical activity [ < 150 or ≥150 min/week based on the total time spent in moderate physical activity each week (11)], medical history (hypertension and diabetes), and medication use at baseline (antihypertensive and lipid lowering drug). The TDI is a composite measure of deprivation based on unemployment, non-car ownership, non-home ownership, and household overcrowding. It is derived from the residential postcode, with a negative value representing high socioeconomic status (12). The data fields used in the UK Biobank study are described in Supplementary Table 1. Missing covariates were imputed using the mode for categorical variables and the median for continuous variables (missing rate < 2.3%).

### Outcome ascertainment

The UKB defines disease phenotypes on the basis of reports from hospital admission records, primary health care information, and death registration records. In our study, the identification of DLBCL cases at baseline and incident cases during follow-up was based entirely on the International Classification of Diseases-10 (ICD-10) code C83. The duration of follow-up was calculated from the baseline date to the earliest date of DLBCL diagnosis, death or the end of follow-up on 2022-10-31, whichever occurred first.

### Statistical analyses

For baseline characteristics, continuous variables are presented as median (interquartile range), and categorical variables as frequency (percentage). In the descriptive analysis of variables, the Wilcoxon rank-sum test was used for continuous variables, and Pearson's Chi-squared test for categorical variables to examine differences between groups.

Cumulative incidence curves were plotted to assess the relationship between serum lipid quartiles and the risk of DLBCL during the follow-up period, with statistical evaluation performed using the log-rank test. The quartile cutpoints were: ApoA (0.42, 1.35, 1.51, 1.70, 2.50); ApoB (0.40, 0.86, 1.02, 1.18, 2.00); HDL (0.25, 1.17, 1.40, 1.67, 3.47); LDL (0.28, 2.95, 3.52, 4.11, 7.96); TC (1.50, 4.91, 5.64, 6.40, 11.80); TG (0.26, 1.05, 1.48, 2.14, 11.28) (Supplementary Figure 2). The Cox proportional risk model was used to determine the hazard ratios (HR) and 95% CI between serum lipid (continuous and quartiles) and DLBCL risk. Three models were used: Model 1 was an unadjusted crude model; Model 2 was adjusted for age, sex, and ethnicity; and Model 3 was further adjusted for BMI, TDI, education level, smoking status, alcohol consumption, physical activity, history of diabetes, history of hypertension, and medication use. To control for multiple comparisons arising from the simultaneous testing of different lipid measures, we applied the Benjamini–Hochberg method to perform false discovery rate (FDR) correction on the *P*-values from the associations of the six core lipid measures (as continuous variables) in Model 3, FDR < 0.05 is considered to be statistically significant.

To determine whether there is a non-linear dose-response relationship between serum lipids and DLBCL risk, restricted cubic spline (RCS) curves were used. This analysis was conducted in R using the “rcs” command, adjusted for the same set of covariates as outlined for the Cox regression models. The analysis utilized 4 degrees of freedom, corresponding to 3 internal knots at equally spaced percentiles.

Subgroup analyses were conducted based on baseline characteristics (age, sex, ethnicity, BMI, education level, history of hypertension, and use of antihypertensive and lipid-lowering medications). Cox regression models were used to examine the association between serum lipid characteristics and DLBCL within different subgroups. The models included interaction terms between these stratifying factors and serum lipid concentrations to explore potential moderating effects on risk factors on a multiplicative scale.

Temporal trajectories were analyzed to observe the dynamic evolution of serum lipids over the 10 years preceding DLBCL diagnosis. A nested case-control study design was employed for this purpose. All cases diagnosed with DLBCL during the follow-up period were considered as cases, and the nested controls were selected through incidence density sampling from cohort members who remained free of DLBCL during the follow-up period. The control group was matched to the cases by age (±2 years), sex, and BMI in a 5:1 ratio. The observation date for the nested controls was set to match that of their corresponding DLBCL cases. Locally weighted scatterplot smoothing (LOWESS) curves were used to plot the mean concentrations of lipids over time leading up to the index date for both cases and controls. Mann-Kendall trend tests were performed to assess the presence of monotonic trends in lipid levels over time between those who developed DLBCL and those who did not.

A two-tailed significance level of p < 0.05 was considered statistically significant. All analyses were performed using RStudio Version 4.5.0. The “survival” and “survminer” packages were used for survival analyses and visualization. The “rms” package was utilized for restricted cubic spline analyses. The Mann-Kendall trend tests were performed using the “Kendall” package. Data visualization and management were supported by “ggplot2” and “dplyr” packages.

## Result

### Baseline characteristics of participants

The baseline characteristics of the study participants are shown in Table 1. A total of 339,172 participants were included in our study. Over a follow-up period of up to 13.8 years, 1,207 participants were diagnosed with DLBCL. Individuals with DLBCL were typically older, predominantly male, and mostly white. In addition, these individuals had higher BMI, lower education levels, and were more likely to have a history of hypertension, use of lipid lowering and antihypertensive medications.

**Table 1:** Baseline characteristics of participants included in the study.

**Characteristic**	**Healthy controls (*N* = 337,965)**	**DLBCL (*N* = 1,207)**	***P***
Age, years			< 0.001
Median (Q1, Q3)	58.00 (50.00, 63.00)	62.00 (57.00, 66.00)	
Gender			< 0.001
Female	182,247.00 (53.92%)	523.00 (43.33%)	
Male	155,718.00 (46.08%)	684.00 (56.67%)	
Ethnicity			< 0.001
Non-White	19,513.00 (5.77%)	40.00 (3.31%)	
White	318,452.00 (94.23%)	1,167.00 (96.69%)	
Drinking status			0.113
Current	310,492.00 (91.87%)	1,097.00 (90.89%)	
Never	15,300.00 (4.53%)	53.00 (4.39%)	
Previous	12,173.00 (3.60%)	57.00 (4.72%)	
Smoking status			0.144
Current	35,604.00 (10.53%)	125.00 (10.36%)	
Never	186,489.00 (55.18%)	636.00 (52.69%)	
Previous	115,872.00 (34.29%)	446.00 (36.95%)	
Townsend deprivation index			0.562
Median (Q1, Q3)	−2.15 (-3.65, 0.51)	−2.15 (-3.77, 0.55)	
Body Mass Index			0.008
Normal	110,129.00 (32.59%)	352.00 (29.16%)	
Obese	80,975.00 (23.96%)	314.00 (26.01%)	
Overweight	145,123.00 (42.94%)	540.00 (44.74%)	
Underweight	1,738.00 (0.51%)	1.00 (0.08%)	
Education			0.001
College	109,972.00 (32.54%)	352.00 (29.16%)	
Other levels	166,521.00 (49.27%)	591.00 (48.96%)	
Unknown	61,472.00 (18.19%)	264.00 (21.87%)	
Physical activity			0.787
Non	125,455.00 (37.12%)	443.00 (36.70%)	
Yes	212,510.00 (62.88%)	764.00 (63.30%)	
Diabetes			0.115
Yes	6,921.00 (2.05%)	33.00 (2.73%)	
No	331,044.00 (97.95%)	1,174.00 (97.27%)	
Hypertension			< 0.001
Yes	25,896.00 (7.66%)	138.00 (11.43%)	
No	312,069.00 (92.34%)	1,069.00 (88.57%)	
Lipid lowering drug			< 0.001
Yes	35,585.00 (10.53%)	177.00 (14.66%)	
No	302,380.00 (89.47%)	1,030.00 (85.34%)	
Antihypotensive_drugs			< 0.001
Yes	37,333.00 (11.05%)	206.00 (17.07%)	
No	300,632.00 (88.95%)	1,001.00 (82.93%)	

DLBCL, Diffuse large B-cell lymphoma.

#### Association between lipid traits and the risk of DLBCL

**Figure 1:**
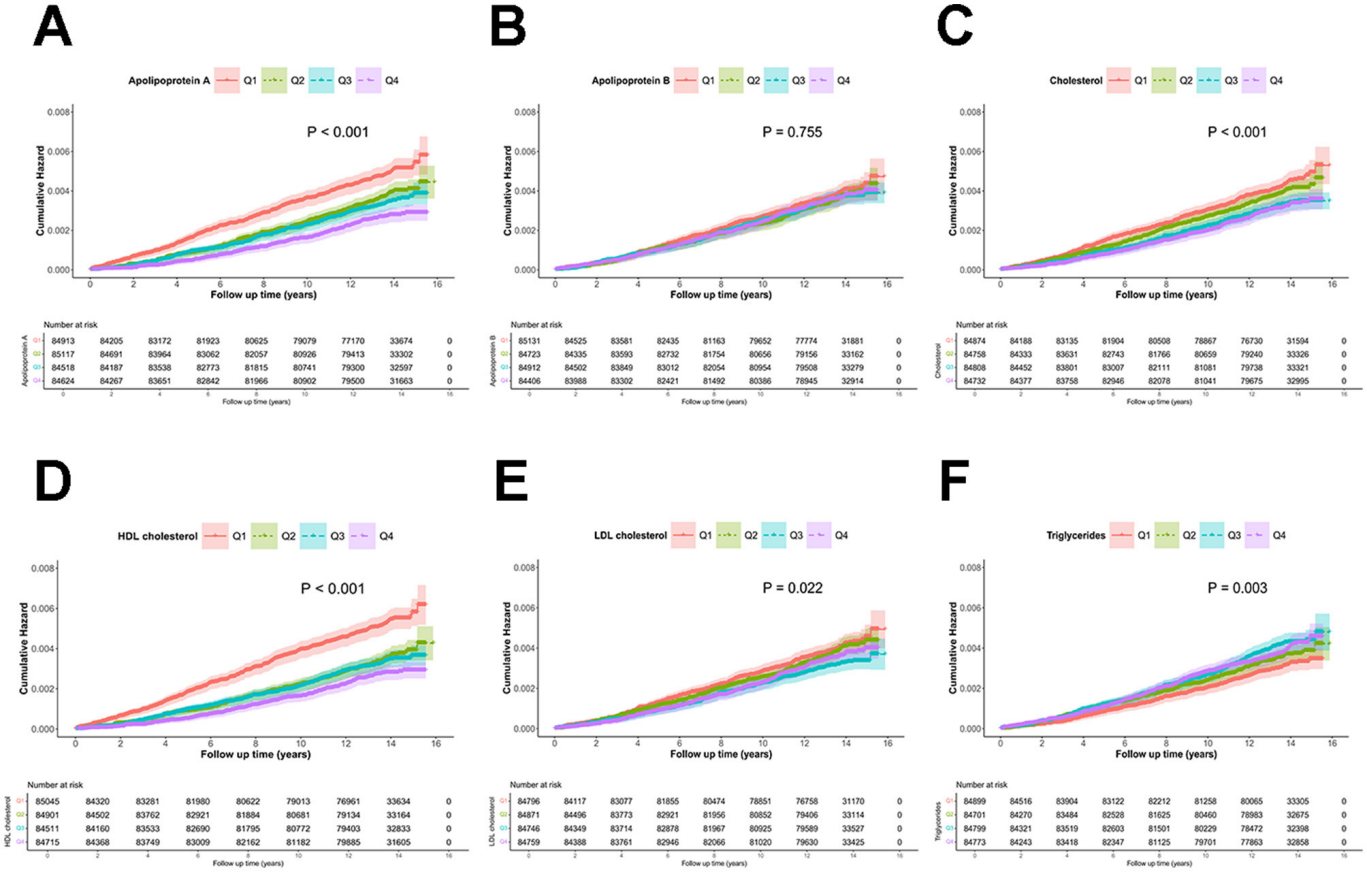
Cumulative incidence of DLBCL stratified by serum lipid levels: apolipoprotein A **(A)**, apolipoprotein B **(B)**, total cholesterol **(C)**, high-density lipoprotein **(D)**, low-density lipoprotein (E), and triglycerides **(F)**.

The risk of DLBCL across different groups (categorized by lipid profile quartiles) was analyzed using cumulative incidence curves. As shown in Figure 1, patients with lower levels of ApoA, TC, HDL, and LDL had a significantly increased risk of DLBCL, while those with higher TG levels also exhibited a significantly higher risk of DLBCL (*P* < 0.05). However, no significant association was found between ApoB and DLBCL risk.

To comprehensively evaluate the relationship between lipid traits and the risk of DLBCL, we developed three Cox regression models (Table 2). In the unadjusted Cox regression analysis (Model 1), low levels of ApoA, HDL, TC and high TG levels were significantly associated with an increased risk of DLBCL. These associations remained significant after adjusting for age, sex, and ethnicity, with the exception of TG (Model 2). After further adjustment for all covariates (Model 3), the significant association between ApoA, HDL, TC and DLBCL risk remained.

**Table 2:** Associations between serum lipid and the risk of DLBCL in the UK Biobank.

**Variable**	**Model 1**	***P***	**Model 2**	***P***	**Model 3**	***P***	**FDR**
ApoA (continuous)	0.42 (0.34, 0.53)	< 0.001	0.41 (0.32, 0.53)	< 0.001	0.42 (0.32, 0.54)	< 0.001	0.006
** ApoA quartile**							
Q1	Ref		Ref		Ref		
Q2	0.76 (0.66, 0.88)	< 0.001	0.76 (0.65, 0.88)	< 0.001	0.76 (0.66, 0.89)	< 0.001	
Q3	0.69 (0.59, 0.81)	< 0.001	0.69 (0.59, 0.81)	< 0.001	0.70 (0.59, 0.82)	< 0.001	
Q4	0.55 (0.46, 0.64)	< 0.001	0.54 (0.45, 0.65)	< 0.001	0.55 (0.46, 0.66)	< 0.001	
ApoB (continuous)	0.87 (0.68, 1.10)	0.246	0.86 (0.68, 1.09)	0.216	0.80 (0.62, 1.03)	0.084	0.101
** ApoB quartile**							
Q1	Ref		Ref		Ref		
Q2	0.93 (0.80, 1.09)	0.383	0.96 (0.82, 1.13)	0.641	0.94 (0.80, 1.11)	0.469	
Q3	0.93 (0.79, 1.08)	0.338	0.93 (0.79, 1.09)	0.371	0.90 (0.76, 1.06)	0.194	
Q4	0.94 (0.80, 1.10)	0.427	0.93 (0.79, 1.08)	0.335	0.88 (0.75, 1.04)	0.146	
HDL (continuous)	0.48 (0.41, 0.57)	< 0.001	0.52 (0.43, 0.63)	< 0.001	0.51 (0.42, 0.62)	< 0.001	0.012
** HDL quartile**							
Q1	Ref		Ref		Ref		
Q2	0.66 (0.57, 0.77)	< 0.001	0.68 (0.59, 0.79)	< 0.001	0.68 (0.58, 0.79)	< 0.001	
Q3	0.63 (0.54, 0.73)	< 0.001	0.67 (0.57, 0.79)	< 0.001	0.67 (0.57, 0.79)	< 0.001	
Q4	0.52 (0.44, 0.61)	< 0.001	0.56 (0.47, 0.67)	< 0.001	0.56 (0.46, 0.67)	< 0.001	
LDL (continuous)	0.91 (0.85, 0.97)	0.005	0.93 (0.87, 1.00)	0.037	0.91 (0.84, 0.97)	0.008	0.012
** LDL quartile**							
Q1	Ref		Ref		Ref		
Q2	0.95 (0.82, 1.11)	0.540	1.05 (0.90, 1.22)	0.555	1.01 (0.86, 1.18)	0.930	
Q3	0.79 (0.67, 0.92)	0.003	0.84 (0.71, 0.99)	0.035	0.80 (0.67, 0.95)	0.010	
Q4	0.89 (0.76, 1.04)	0.128	0.92 (0.79, 1.08)	0.308	0.87 (0.73, 1.03)	0.108	
TC (continuous)	0.88 (0.83, 0.92)	< 0.001	0.90 (0.86, 0.95)	< 0.001	0.88 (0.83, 0.93)	< 0.001	0.012
** TC quartile**							
Q1	Ref		Ref		Ref		
Q2	0.90 (0.78, 1.05)	0.191	1.01 (0.87, 1.18)	0.884	0.97 (0.82, 1.13)	0.676	
Q3	0.75 (0.64, 0.88)	< 0.001	0.82 (0.70, 0.96)	0.015	0.78 (0.65, 0.92)	0.003	
Q4	0.73 (0.63, 0.86)	< 0.001	0.78 (0.65, 0.91)	0.002	0.73 (0.61, 0.87)	< 0.001	
TG (continuous)	1.07 (1.01, 1.12)	0.022	1.00 (0.94, 1.06)	0.97	0.98 (0.92, 1.04)	0.564	0.564
** TG quartile**							
Q1	Ref		Ref		Ref		
Q2	1.15 (0.98, 1.37)	0.093	0.97 (0.82, 1.15)	0.755	0.96 (0.81, 1.13)	0.610	
Q3	1.34 (1.14, 1.57)	< 0.001	1.05 (0.89, 1.24)	0.552	1.02 (0.86, 1.20)	0.845	
Q4	1.26 (1.07, 1.49)	0.006	0.98 (0.83, 1.15)	0.790	0.93 (0.78, 1.11)	0.431	

ApoA, apolipoprotein A; ApoB, apolipoprotein B; HDL, high-density lipoprotein cholesterol; LDL, low-density lipoprotein cholesterol; TC, total cholesterol; TG, triglycerides. Model 1 was an unadjusted crude model; Model 2 was adjusted for age, sex, and ethnicity; and Model 3 was further adjusted for BMI, TDI, education level, smoking status, alcohol consumption, physical activity, history of diabetes, history of hypertension, and medication use.

**Figure 2:**
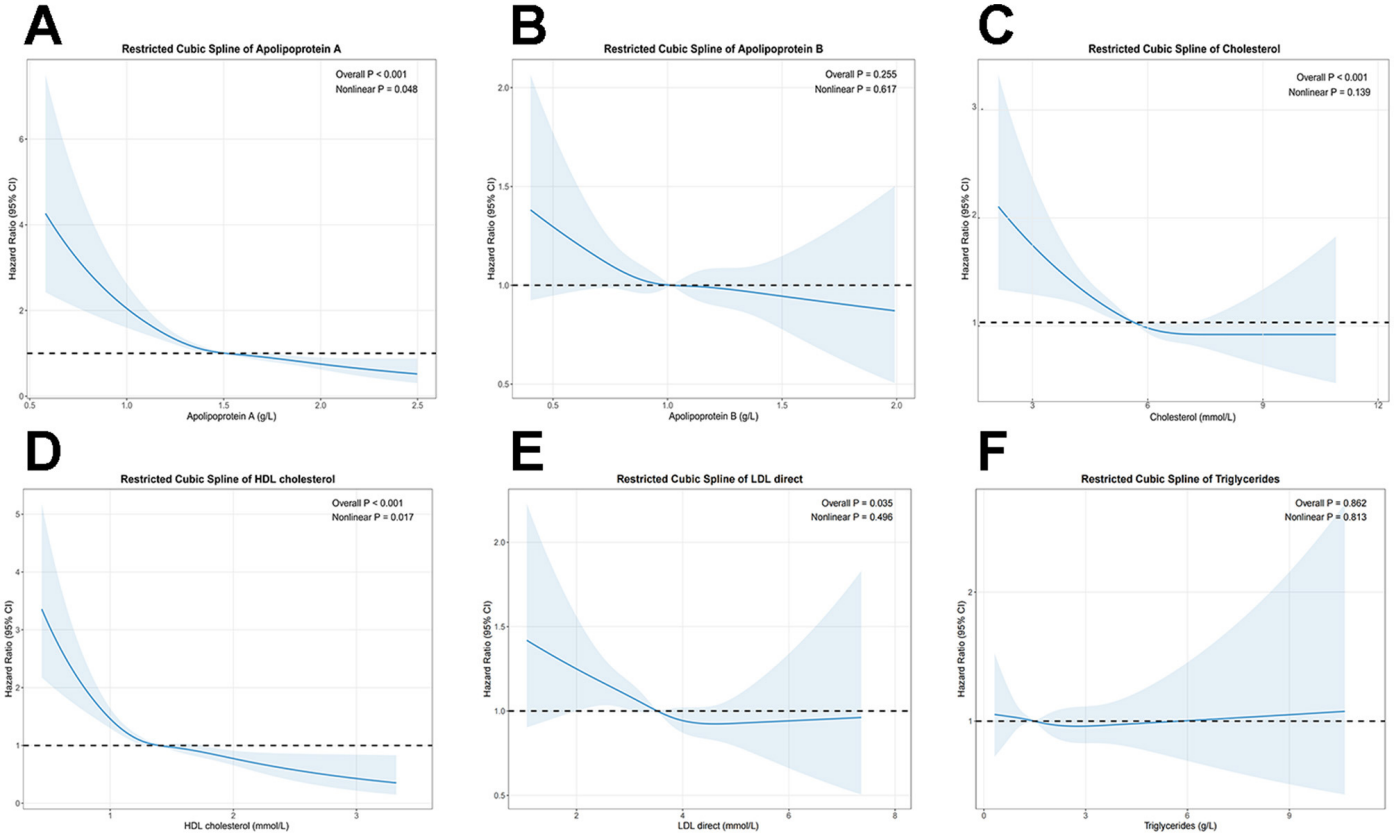
Restricted cubic spline models depicting the dose-response relationship of serum lipids with the risk of DLBCL. The analysis is presented for apolipoprotein A **(A)**, apolipoprotein B **(B)**, total cholesterol **(C)**, high-density lipoprotein (HDL) **(D)**, low-density lipoprotein (LDL) **(E)**, and triglycerides **(F)**. All models were adjusted for age, sex, ethnicity, BMI, TDI, education level, smoking status, alcohol consumption, physical activity, history of diabetes, history of hypertension, and medication use.

Multivariable-adjusted RCS analysis indicated a non-linear relationship between ApoA, HDL, and the incidence of DLBCL (non-linear *p*-values of 0.048 and 0.017, respectively). In contrast, TC was linearly negatively associated with DLBCL incidence, with a non-linear *p*-value of 0.139 (Figure 2).

### Subgroup analysis

**Figure 3:**
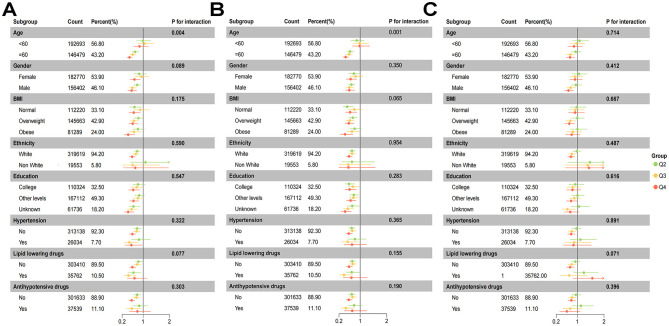
Subgroup analyses for the association between serum lipid levels and DLBCL risk. Results are presented for apolipoprotein A **(A)**, high-density lipoprotein (HDL) **(B)**, and total cholesterol **(C)**.

To further assess the heterogeneity in the association between lipid traits and the risk of DLBCL, we conducted stratified subgroup analyses and interaction tests (Figure 3 and Supplementary Table 2). ApoA and HDL were significantly associated with DLBCL risk in most subgroups. Furthermore, interaction analyses revealed a significant effect modification by age for both ApoA (P for interaction = 0.004) and HDL (P for interaction = 0.001).

### Pre-DLBCL trajectories for lipid traits

**Figure 4:**
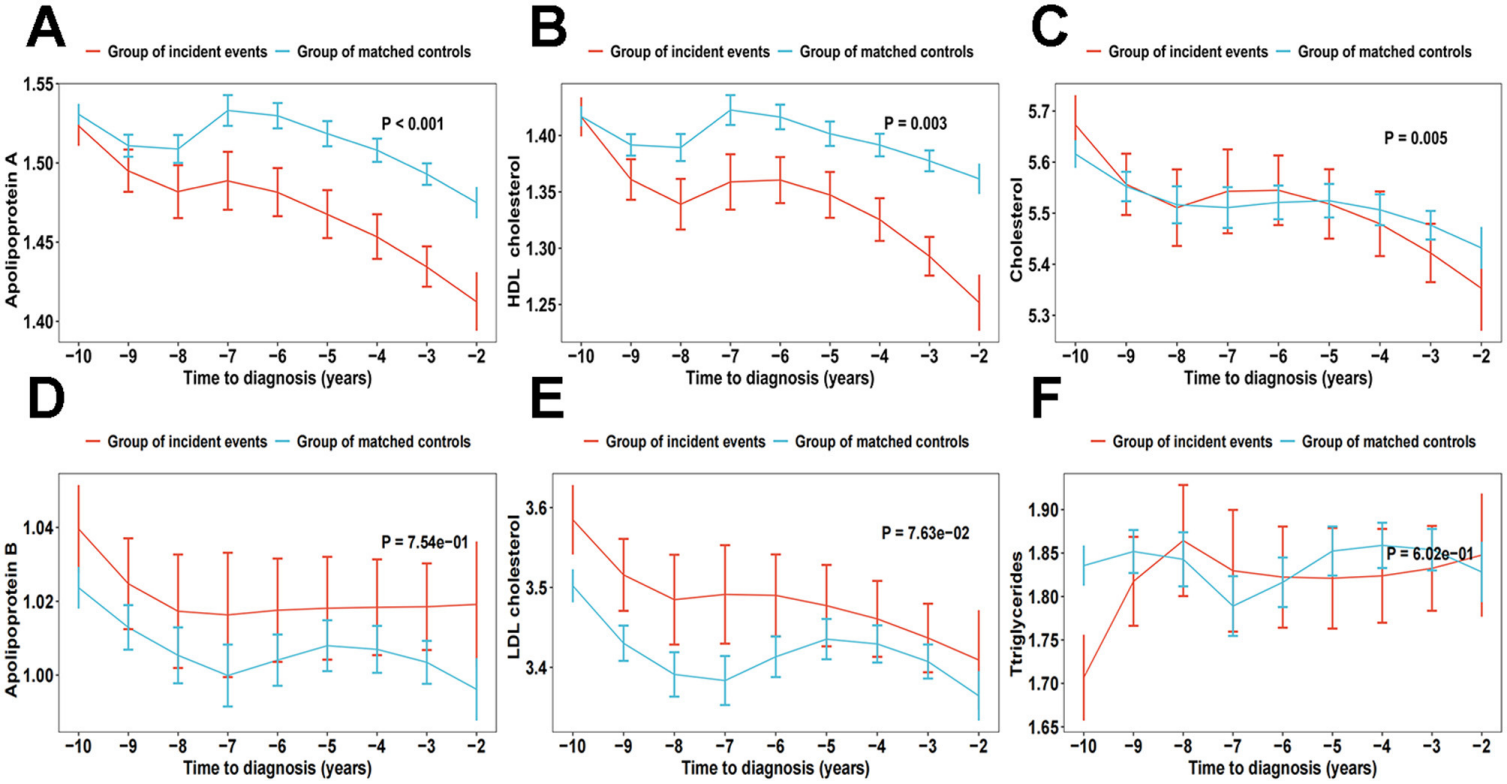
Trajectories of serum lipid levels prior to DLBCL diagnosis for apolipoprotein A **(A)**, high-density lipoprotein **(B)**, total cholesterol **(C)**, apolipoprotein B **(D)**, low-density lipoprotein **(E)**, and triglycerides **(F)**.

Using a 10-year retrospective timescale starting from the diagnosis of DLBCL, we meticulously tracked the evolution of lipid traits and compared them to individuals who did not develop DLBCL during the same period. The smoothed spline curves revealed that, compared to individuals without DLBCL, those who developed DLBCL exhibited declining levels of ApoA and HDL over time, with a notably steeper decline in the 5 years preceding disease onset. Although TC levels showed significant differences in the trajectories between the two groups, its trajectory was more complex, exhibiting varying patterns across different stages. However, no similar trends were observed for other lipid traits (Figure 4).

## Discussion

In this large prospective cohort study based on the UK Biobank, we systematically evaluated the association between serum lipid traits and the risk of DLBCL. Our findings demonstrated that lower levels of ApoA, HDL, and TC were significantly associated with an increased risk of DLBCL in multivariable-adjusted Cox regression models and RCS analysis. Notably, longitudinal trajectory analyses also revealed a marked decline in ApoA and HDL levels in DLBCL cases during the years preceding diagnosis, particularly within the last 5 years. These findings suggest that certain lipid traits may play an important role in the pathogenesis or early development of DLBCL.

Our findings are consistent with previous studies, which have demonstrated a close association between lipid metabolism dysregulation and the development of lymphoma (8, 13–15). HDL and ApoA are key components of reverse cholesterol transport and possess various biological functions, including anti-inflammatory, antioxidant, and immune-regulatory effects (16). Previous studies have confirmed a negative correlation between ApoA and HDL levels and the risk of various cancers, including lung, prostate, liver, and colorectal cancers (17, 18). Although prior research suggests that subclinical tumor activity can alter lipid metabolism, and thus reverse causality cannot be entirely ruled out (19), our prospective cohort study provides compelling evidence to the contrary. We observed a negative relationship between ApoA/HDL levels and DLBCL risk, with a significant decline in these biomarkers beginning up to a decade before diagnosis. This prolonged preclinical trajectory strongly indicates that alterations in lipid metabolism precede and may contribute to the development of DLBCL.

Although the mechanisms underlying the relationship between HDL/ApoA and DLBCL risk are not yet fully understood, several potential explanations exist. Existing evidence suggests that the HDL/ApoA system exerts its potential tumor-suppressive effects through several mechanisms, including mediating reverse cholesterol transport via ABCA1/ABCG1, which reduces the cholesterol content in tumor cell membranes and disrupts lipid raft integrity (20); regulating immune cell function in the tumor microenvironment by promoting the polarization of tumor-associated macrophages to the M1 phenotype, inhibiting the activation of pro-inflammatory signaling pathways such as NF-κB/STAT3, and enhancing the anti-tumor activity of CD8+ T cells (16, 21); and clearing reactive oxygen species through its antioxidant enzymes, such as paraoxonase 1, thereby alleviating DNA damage induced by oxidative stress (22).

Cholesterol, an essential component of cell membranes, plays a key role in cell signaling and immune function regulation. Our study suggests that an increased TC levels may reduce the risk of DLBCL, a finding that contradicts previous research (3). This discrepancy may be due to cholesterol in the bloodstream modulating immune cells to exert anti-tumor effects, while intracellular cholesterol in tumor cells could activate oncogenic pathways, promoting tumor development (23). TG are often associated with insulin resistance, hyperleptinemia, and enhanced lipolysis. These metabolic disturbances can activate pathways such as STAT3 and MAPK, promoting B cell proliferation and angiogenesis (24). In our study, the positive correlation between TG and DLBCL was significant in the unadjusted model; however, after adjusting for covariates, this association was no longer statistically significant, suggesting the potential influence of mediating or confounding factors. Cox regression analysis did not show a significant association between ApoB and LDL levels and DLBCL risk, which is inconsistent with some earlier studies (8). This discrepancy may be attributed to several factors. First, variations in study populations, lipid measurement methods (including potential variability from non-fasting samples), or residual confounding (e.g., by statin use despite adjustment) could contribute. More importantly, the unique metabolic profile of DLBCL, as a hematologic malignancy, might differ from that of solid tumors. Subgroup analyses were conducted to explore the consistency of the associations across different populations. Notably, the significant protective associations of ApoA and HDL observed in the overall cohort were attenuated and not statistically significant in some subgroups, such as individuals under 60 years old, non-white participants, or those using lipid-lowering/antihypertensive medications. The lack of significance in these subgroups is likely attributable to limited statistical power due to smaller sample sizes and potential residual confounding, rather than constituting robust evidence of true biological heterogeneity. While the observed statistical interaction with age warrants further investigation, it does not confirm a differential biological effect. Similarly, the association between TC and DLBCL risk, while significant overall, was not consistent across all subgroups. Therefore, future studies with larger, diverse cohorts and more detailed phenotypic data are necessary to conclusively establish whether demographic or clinical factors modify the relationships between lipid traits and DLBCL risk. Trajectory analysis provides new insights into the temporal dynamics of lipid changes prior to the diagnosis of DLBCL. We found that ApoA and HDL levels showed a significant decline over the 10 years preceding diagnosis, with a sharper decline in the 5 years before diagnosis. This may reflect a compensatory response of the body to early malignant processes or systemic inflammation (25). Although the trend in TC showed statistical significance, its complex trajectory suggests that TC's role in DLBCL progression may not be as direct as anticipated or may be more susceptible to confounding factors. The temporal changes in ApoB, LDL, and TG were not statistically significant (*P* > 0.05), suggesting that their response to the early progression of DLBCL is relatively weak.

This study is a prospective, large-scale cohort study that is the first to evaluate the association between lipid profiles and DLBCL. However, several limitations exist. First, the study is based on data from the UK Biobank, with participants primarily of European descent aged 40–69 years, so the findings may not be generalizable to other age groups, ethnicities, or socioeconomic backgrounds. Second, although we adjusted for multiple potential confounders, unmeasured factors such as genetic background, dietary habits, and lifestyle may still influence the relationship between lipid metabolism and DLBCL. Additionally, lipid data were only collected at baseline, and although we analyzed lipid trajectories over the 10 years prior to diagnosis, the lack of dynamic monitoring limits the accurate assessment of metabolic changes. As an observational study, there is a lack of animal or cellular experimental data to support this hypothesis.

## Conclusion

In conclusion, our study provides compelling evidence that low levels of ApoA, HDL and TC are associated with an increased risk of DLBCL. Longitudinal lipid trajectories suggest that changes in lipid metabolism may occur several years prior to DLBCL diagnosis, offering potential opportunities for early detection or intervention. Future research should explore the biological mechanisms underlying these associations and evaluate whether lipid-modulating strategies can reduce the risk of DLBCL in high-risk populations.

## Data Availability

The original contributions presented in the study are included in the article/Supplementary material, further inquiries can be directed to the corresponding author.

## Supplementary material

Supplementary Figure 1 The flow diagram of participant inclusion and exclusion.

Supplementary Figure 2 Distribution of serum lipid.

Supplementary Table 1 Extraction of data variables from the UK Biobank.

Supplementary Table 2 Subgroup analyses of the association between the serum lipid and the risk of DLBCL.
