# Supplementary figures and images for: Associations of serum lipid traits with DLBCL: a prospective cohort study from the UK Biobank
Source: Front Nutr. 2026 Feb 10;13:1707450. doi: 10.3389/fnut.2026.1707450 (PMC12929521; doi:10.3389/fnut.2026.1707450)

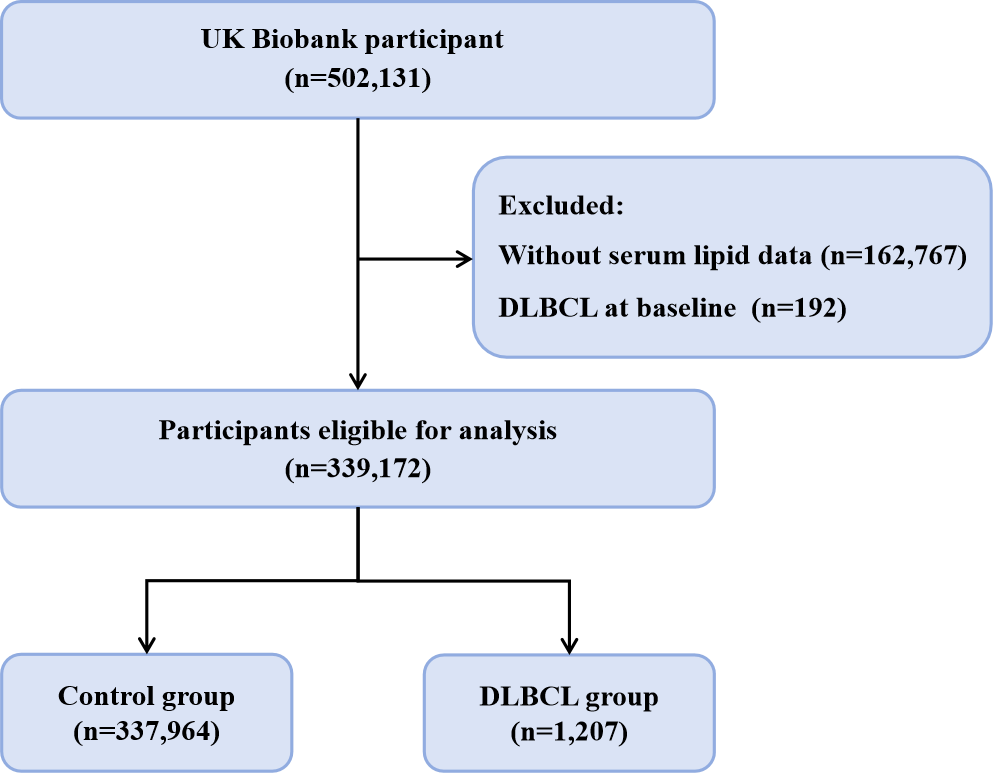

Supplement: Supplementary Figure 1 — The flow diagram of participant inclusion and exclusion. [file Image_1.png]

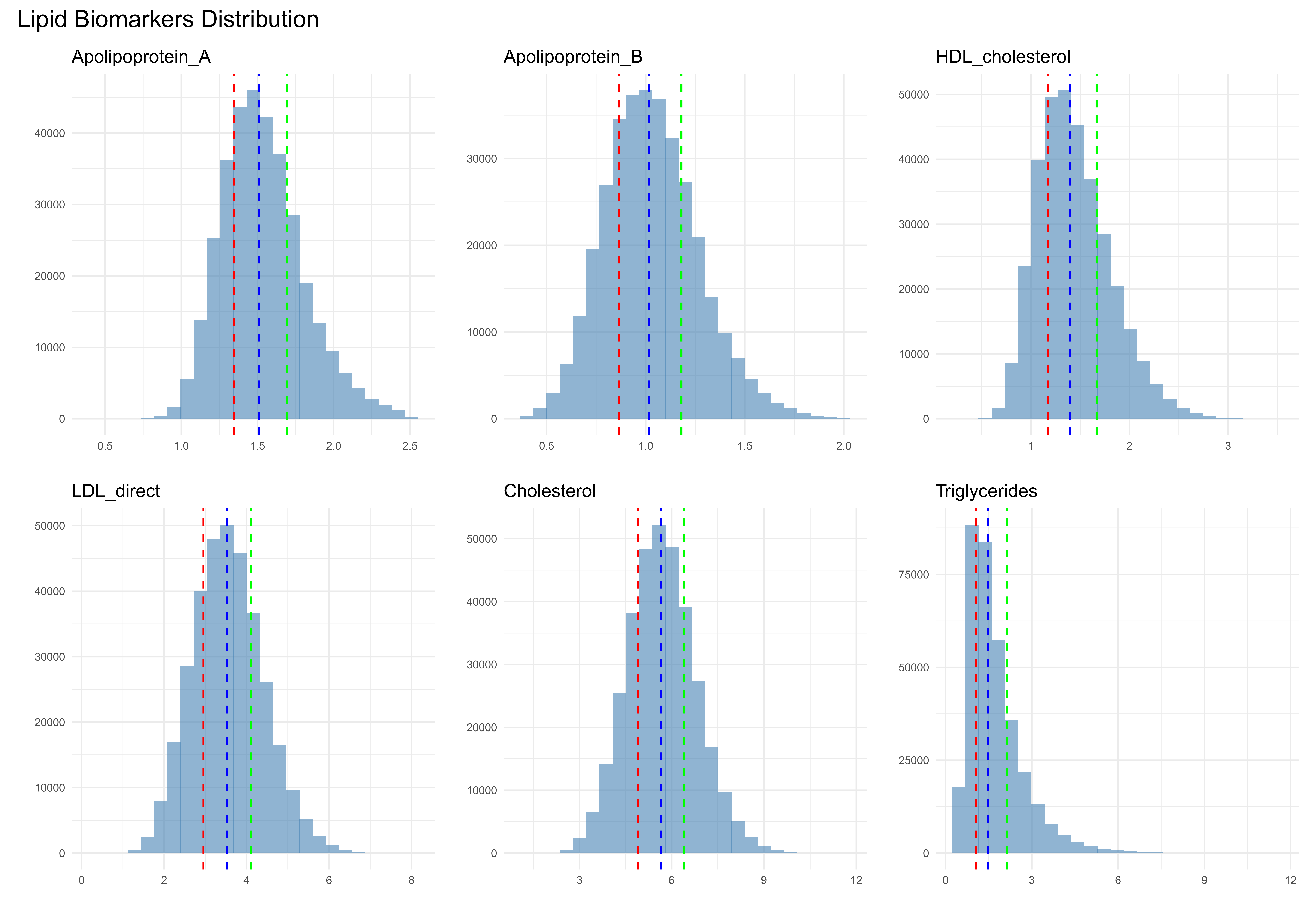

Supplement: Supplementary Figure 2 — Distribution of serum lipid. [file Image_2.png]
